# Latest development on RNA-based drugs and vaccines

**DOI:** 10.4155/fsoa-2017-0151

**Published:** 2018-05-04

**Authors:** Kenneth Lundstrom

**Affiliations:** 1 PanTherapeutics, Route de Lavaux 49, CH1095 Lutry, Switzerland

**Keywords:** mRNA drugs, RNA-based drugs, RNA-based vaccines, self-amplifying RNA

## Abstract

Drugs and vaccines based on mRNA and RNA viruses show great potential and direct translation in the cytoplasm eliminates chromosomal integration. Limitations are associated with delivery and stability issues related to RNA degradation. Clinical trials on RNA-based drugs have been conducted in various disease areas. Likewise, RNA-based vaccines for viral infections and various cancers have been subjected to preclinical and clinical studies. RNA delivery and stability improvements include RNA structure modifications, targeting dendritic cells and employing self-amplifying RNA. Single-stranded RNA viruses possess self-amplifying RNA, which can provide extreme RNA replication in the cytoplasm to support RNA-based drug and vaccine development. Although oligonucleotide-based approaches have demonstrated potential, the focus here is on mRNA- and RNA virus-based methods.

## Lay abstract

Drug development has suffered from inefficiency, side effects and high costs. For this reason novel approaches for drug discovery are of great importance. RNA-based methods provide the advantage of targeting ‘production’ of drugs to diseased cells and vaccines to immune response-stimulating cells. RNA drugs have demonstrated therapeutic efficacy in eye and heart diseases and in various cancers in clinical trials. Likewise, RNA-based vaccines have provided protection against challenges with lethal doses of viruses such as Ebola and cancer cells in animal models.

Modern drug discovery has suffered from finding improved and/or novel drugs due to inefficient delivery issues, drug efficacy and safety. In addition to classic approaches developing small molecule-based drugs and biotherapeutics, employing nucleic acids for therapeutic applications have become of potential interest [1,2]. In this context, plasmid DNA and oligonucleotide drugs have been studied. However, recent technology improvements have presented serious opportunities for employing RNA-based approaches [3]. The straightforward approach involves administration of mRNA with the goal of providing immediate translation in target cells resulting in expression of therapeutic genes for treatment of disease or antigens in the case of vaccine development. Due to the presence of RNAses, the delivered transcripts are subjected to rapid degradation resulting in restricted transient expression and compromising the efficacy of treatment [4]. Major efforts have been dedicated to improve RNA stability [5]. Similarly, attention has been paid to delivery issues including encapsulation of RNA molecules consisting of lipids, polymers and nanoparticles [6] as well as targeting dendritic cells (DCs) known as antigen-presenting cells [7]. In another approach self-amplifying RNA molecules based on RNA viruses have been applied for delivery of RNA [8]. In this review, methods for RNA stabilization, delivery and amplification are presented. Moreover, the latest development on RNA-based drugs and vaccines are discussed. The focus here, will be on mRNA- RNA virus-based drugs and vaccines and oligonucleotide-based approaches will not be discussed.

## RNA stability improvements

Due to the sensitivity of single-stranded RNA (ssRNA) to degradation multiple efforts have been made to improve the stability of RNA molecules [5]. These include engineering of sequences which have been demonstrated to stabilize mRNA and in some cases to contribute to the initiation of translation. Moreover, chemical modifications of nucleosides have also provided improved resistance to degradation.

### Cap analog

The 5′ 7-methylguanosine triphosphate (m^7^G) Cap plays an important role in RNA stability [9]. Although cap analogs have been applied for *in vitro* transcription of RNA a problem has been the incorporation of cap analogs in reverse orientation resulting in an inability to efficiently transcribe mRNAs [10,11]. Design of antireverse cap analogs (ARCAs) with only one 3′-OH group instead of two 3′-OH groups present in cap analogs prevented the incorporation in reverse orientation [12]. Application of ARCAs provides more than the double RNA transcription efficiency compared with conventional cap analogs. Moreover, protein expression duration and levels have been shown to be improved in cells transfected with ARCA-capped *in vitro* transcribed RNA [13]. Due to less than 100% efficiency in the capping procedure methods for post-transcriptional cap addition have been developed to improve the translation of uncapped RNA [10].

It was recently discovered that some bacterial RNA species possess a 5′-end structure similar to cap in eukaryotic RNA [14]. Particularly, 5′-end nicotinamide adenine dinucleotide (NAD^+^) and 3′-dephospho-coenzyme A (dpCoA) have been found in both Gram-negative and -positive bacteria. NAD^+^, reduced NAD^+^ (nicotine adenine dinucleotide hydogen, NADH) and dpCoA have been postulated to be added to RNA after initiation of transcription similar to cap analogs [15]. However, a more recent study demonstrated that NAD^+^, NADH and dpCoA are incorporated into RNA during the transcription initiation phase [16]. They were shown to serve as noncanonical initiating nucleotides (NCINs) for *de novo* transcription initiation by cellular RNA polymerase (RNAP). Furthermore, both bacterial RNAP and eukaryotic RNAP II incorporate NCIN caps. The efficiency of NCIN capping is related to the promoter DNA sequences at and upstream of the transcription initiation site. Additionally, NCIN capping takes place *in vivo* with functional consequences. These findings might be important in future attempts to stabilize RNA for optimized delivery and expression.

### Poly(A) tail

Another approach to stabilize RNA molecules is by engineering the poly(A) tail at the 3′ end of mRNAs [10]. It has been demonstrated that poly(A) tails work synergistically with 5′ m^7^G cap sequences by binding the PABP [17]. PABP has been shown to interact with the eukaryotic translation initiation factor eIF4G, which then forms a complex with the 5′ m7G cap and the eukaryotic translation initiation factor eIF4E [18]. Poly(A) tails can be engineered to mRNAs by encoding the poly(A) tail on the DNA template or using recombinant poly(A) polymerase to extend *in vitro* transcribed RNA after transcription. The drawback of applying recombinant poly(A) polymerase is the generation of poly(A) tails of various lengths. In contrast, as mRNAs transcribed from DNA templates generate poly(A) tails of a defined length it is the preferred approach [19].

In the context of poly(A) tail engineering, it has been demonstrated that the increase in poly(A) tail length generates enhanced efficiency of polysome formation, which also impacts the level of protein expression [20]. Based on several studies it has been concluded that the optimal length of the poly(A) tail for mRNA *in vitro* transcription is between 120 and 150 nucleotides [19,21–22].

### 5′ and 3′ end untranslated regions

Untranslated regions (UTRs) have been demonstrated to play important roles in post-transcriptional regulation of gene expression. These include modulation of mRNA transport from the nucleus and translation efficiency [23], subcellular localization [24] and mRNA stability [25]. Moreover, UTRs, particularly the conserved stem-loop structure in the 3′ end UTR and the selenocysteine insertion sequence element, are also responsible for the incorporation of selenocysteine at UGA codons of mRNA providing encoding of selenoproteins [26,27].

The introduction of UTRs from the 5′ and 3′ ends can optimize *in vitro* transcription of mRNA due to the presence of important regulatory elements. Incorporation of *alpha-globin* 3′ end UTRs has been demonstrated to stabilize mRNA [28,29]. Moreover, *β-globin* 5′ end and 3′ end UTRs provide improved translation efficiency [23–25]. *Globin* UTRs have been applied for optimizing RNA for *in vitro* transcription followed by RNA electroporation of autologous T cells [30] and intranodal injection of naked antigen-encoding RNA [31]. Additionally, DCs transfected with antigen-expressing UTR-optimized RNA have been used for immunization of CMV-seropositive individuals and cancer patients [32].

### Chemically modified nucleosides

In attempts to improve the therapeutic properties of RNA, incorporation of natural nucleosides during RNA post-translational processing has proven useful for providing reduced immunogenicity of *in vitro* transcribed RNA [33]. For instance, *in vitro* transcribed mRNA containing modified pseudouridine showed enhanced RNA stability and translation [34,35]. However, although RNA can stimulate the immune system by activation of Toll-like receptors (TLRs), incorporation of modified nucleosides (methylated or pseudouridine) decreased the activity, which resulted in significantly lower cytokine levels and biomarker activity in DCs [34]. This approach therefore prevents recognition by TLR3, TLR7 and TLR8 and inducing immune responses against the *in vitro* transcribed RNA [35]. Furthermore, in attempts to increase and prolong mRNA translation, high performance liquid chromatography purification of *in vitro* transcribed mRNA was applied to remove dsRNA contaminants, which resulted in reduced production of type 1 IFN and pro-inflammatory cytokines [36]

### RNA delivery

The difficulties in achieving efficient delivery of RNA have seriously hampered the application of RNA for drug and vaccine development. For this reason, a variety of approaches have been evaluated for improved RNA delivery including optimized injection strategies, gene gun-based administration, protamine condensation, RNA adjuvants and encapsulation of RNA in nanoparticles consisting of polymers and liposomes [10].

### Naked RNA

The simplest application comprises of administration of naked mRNA by intramuscular injection, originally demonstrated by *in vivo* reporter gene expression in mice [37]. Furthermore, immunization with carcinoembryonic antigen (CEA) mRNA showed anti-CEA antibody responses in mice confirmed the feasibility of administration of naked RNA [38]. Although a number of additional studies in animal models showed elicited antibody and T cell responses [39,40], but the rapid RNA degradation by ubiquitous RNases indicated limitations of this approach [41,42].

Several approaches have been evaluated to improve delivery including direct injection into the cytoplasm by application of gene gun-based delivery [43]. *In vitro* transcribed mRNA coated in gold particles can penetrate cell membranes [44]. In a mouse model gene gun-based mRNA for *melanocyte self-antigen TRP2* linked to *EGFP* (enhanced green fluorescent protein) elicited antigen-specific cellular and humoral responses and provided protection against B16 melanoma lung metastases [39]. Another approach has been protamine condensation of mRNA, which provides protection against RNA degradation and stimulation of immune responses through MyD88, TLR7 and TLR8 dependent pathways [45–47]. Protamine condensation was demonstrated to stimulate the generation of antigen-specific IgG antibodies and activation of a specific cytotoxic T lymphocyte response [48]. Moreover, intradermal administration of protamine condensed mRNA in patients with metastatic melanoma showed safe delivery and increased vaccine-directed T cells in two of four evaluable patients and a complete response in one of seven patients with measurable disease [49].

### Adjuvants & costimulatory molecules

In many vaccine applications it has been demonstrated that addition of adjuvants can substantially boost immune responses [50]. It has been discovered that naked mRNA can itself act as an adjuvant and thereby stimulate immunogenicity [10]. Other molecules such as protamine, poly I:C RNA and CpG containing motifs can enhance the efficacy of immune responses for mRNA-based vaccines [51]. Another approach has been to incorporate costimulatory molecule sequences such as *CD40L*, *CD70*, O*X40L*, *GITR* and *CD83* into the mRNA to further increase the immunogenicity [52–54].

### Encapsulated RNA & targeting of DCs

In attempts to improve delivery and stability of RNA several encapsulation approaches have been evaluated [55]. In this context, cationic liposomes such as N-[1-(2,3-dioleoloxy)propyl]-N,N,N-trimethyl ammonium chloride 1 (DOTAP) have been applied for RNA encapsulation [56–59]. Nanoparticles have been demonstrated to provide mRNA protection against nuclease degradation and enhanced cellular uptake [60]. Further development has seen the engineering of fully degradable nanoparticles with a pH responsive poly-(b-amino ester) core and a phospholipid shell [61]. Efficient nanoparticle-based *in vivo* mRNA delivery has been achieved generating strong immune response in animal models [62,63]. For instance, *OVA* mRNA molecules encapsulated in DOTAP liposomes were injected intradermally into mouse ear pinnae, which provided protection against subcutaneous tumor challenges with EG7-OVA cells [64]. Potential particle aggregation leading to reduced extracellular and intracellular gene delivery may explain the superior CTL (cytotoxic T lymphocyte) responses of intravenous administration in comparison to intradermal injection. It was also demonstrated that incorporation of the helper lipid DOPE with fusogenic properties provided four-times higher CTL responses for mRNA encapsulated in DOTAP/DOPE in comparison to DOTAP liposomes. Codelivery of *OVA* mRNA with *GM-CSF* mRNA resulted in enhanced CTL responses. In contrast, co-distribution of CD80 and IL-2 did not show this effect. In another approach, histidylated lipids have been optimized for mRNA delivery [65]. Systemic administration of *melanoma-associated antigen MART1* mRNA histidylated lipopolyplexes formulated with a PEGylated derivative of histidylated polylysine and L-histidine-ethylamide liposomes demonstrated specific and significant protection against progression of B16F10 melanoma tumors. Enhanced anti-B16 responses were obtained by using a formulation containing both *MART1* and *MART1-LAMP1* mRNAs.

DCs are professional antigen-presenting cells, which play an important role in stimulation of immune responses [10]. Targeting DCs therefore presents a strategy to enhance immunogenicity *in vivo*. However, early observations indicated that DCs were only poorly transfected by lipoplexes [62]. Nanoparticle formulations have therefore been optimized for enhanced targeting of DCs [66]. Related to cancer immunotherapy, DCs can be transfected with either *tumor-associated antigens (TAAs)* mRNA or total tumor RNA [10]. DCs transfected with *TAA* mRNAs can be applied directly for vaccine strategies without the need of utilizing patient-specific tumor cells or antigens [67–69]. The disadvantages comprise the lack of identified TAAs for many cancers and the selection of TAAs can be difficult as not all identified TAAs elicit antitumor immune responses. A number of studies on *TAA* mRNAs have generated stimulation of antitumor responses [70]. For instance, DCs transfected with *prostate-specific antigen (PSA) TAA* mRNAs were administered to prostate cancer patients, which elicited a PSA-specific T-cell response and a significant decrease in PSA levels in six of seven patients [71]. Moreover, immunization with *CEA* mRNA transfected DCs showed good tolerance in pancreatic cancer patients although antitumor responses were obtained in only six out of 24 patients [72,73]. In another approach, mannosylated histidylated lipopolyplex nanoparticles have been formulated for enhanced mRNA transfection of DCs [66]. Intravenous administration demonstrated four-times more DCs expressing EGFP for mRNA-loaded Man(11)-LPR100 compared with sugar-free LPR100. The improved transfection of DCs correlated with enhanced inhibition of B16F10 melanoma growth and extended survival time after immunization with *MART1* mRNA-loaded Man(11)-LPR100.

The approach of using total tumor RNA from cancer patients has been evaluated in clinical settings for brain [74], lung [75], renal [76,77] cancers and melanoma [78–80]. In this context, clinical responses to brain tumors and neuroblastomas were observed in roughly a third of the enrolled patients [74]. Moreover, studies in patients with renal cell carcinoma displayed no evidence of dose-limiting toxicity or induced autoimmunity [76].

### Self-amplifying RNA

ssRNA viruses have been frequently applied for vaccine development and cancer immunotherapy due to their capacity of RNA self amplification [8]. Among RNA viruses alphaviruses, flaviviruses, rhaboviruses and measles viruses have been engineered as expression and delivery vectors as briefly described below.

Alphaviruses belong to the family of togaviruses, possess a ssRNA genome of positive polarity and contain an envelope structure [81]. The nonstructural genes *nsP1–4*, responsible for highly efficient RNA replication, provides extreme amplification of RNA molecules, which due to the positive polarity can be directly translated in the cytoplasm. Both replication-deficient and -proficient alphavirus vectors have been engineered for gene delivery *in vitro* and *in vivo*. Moreover, the vectors can be applied in various forms such as recombinant viral particles, naked replicon RNA or layered RNA-DNA vectors [8]. Likewise, flaviviruses carry a ssRNA genome with positive polarity, which has allowed their applications in cancer immunotherapy in a similar way to alphaviruses [82]. In contrast, both rhabdoviruses [83,84] and measles [85] viruses possess a genome of negative polarity, which has required different strategies for vector development. However, that has not restricted their employment for vaccine development and immunotherapeutic applications.

Self-amplifying RNA virus vectors have been frequently used for development of vaccines against infectious diseases and cancer [8]. For instance, protection against Ebola challenges has been confirmed in several animal models after immunization with recombinant particles of Kunjin virus (flavivirus) [86], Vesicular stomatitis virus (VSV) (rhabdovirus) [87] and Venezuelan equine encephalitis virus (VEE) (alphavirus) [88] expressing the glycoprotein of Ebola virus. Moreover, vesicular stomatitis virus-glycoprotein particles have been subjected to a Phase I/II clinical trial, which demonstrated good safety and Ebola immunogenicity [89]. Furthermore, immunization with measles virus expressing the envelope protein domain III (ED3) provided protection against Dengue virus in mice [90]. Interestingly, gene silencing approaches applying miRNA sequences have been evaluated for VEE, demonstrating down-regulation of VEE replication in an animal model [91]. In another study, VEE RNA dependent RNA polymerase, a key player in VEE replication, was targeted by five miRNAs of which three showed significant inhibition of VEE replication in BHK cells [92].

Related to cancer immunotherapy, a large number of studies have confirmed that self-amplifying RNA viruses can generate tumor regression, prolonged survival and even tumor protection in animal models for various cancers [8]. For instance, expression of GM-CSF from Kunjin virus particles resulted in tumor regression in a mouse melanoma model [93]. Moreover, VEE particles expressing PSCA (prostate stem cell antigen) demonstrated tumor protection in a prostate cancer model [94]. Prolonged survival of mice with implanted brain tumor xenografts was obtained after SFV-based delivery of neuron-specific miR-124 [95]. Interestingly, a single intramuscular injection of naked *SFV-LacZ* RNA provided tumor protection in mice challenged with CT26 colon tumors [96]. It was recently discovered that immunization with self-amplifying RNA vectors triggered early robust type I IFN and IFN-stimulated responses at the site of injection, which might eventually provide an adjuvant effect or reduced antigen expression [97]. Studies in IFN receptor knock-out mice suggested that minimizing early type I IFN responses can be useful to enhance the primary expression from self-amplifying RNA vectors. Another finding relates to the combination of self-amplifying replicon RNA with nanotechnology [98]. Encapsulation of replicon RNA in chitosan nanoparticles provided efficient delivery to DCs and significantly enhanced induction of immune responses *in vivo*. Furthermore, in attempts to prevent the host immune defense induced by intermediate dsRNA of self-amplifying RNA viruses, nonreplicative mRNAs encoding vaccinia virus immune evasion proteins E3, K3 and B18 were codelivered [99]. This approach provided significant suppression of protein kinase R and IFN pathway activation and enhanced the expression of self-amplifying RNA and improved delivery.

Furthermore, a Phase I clinical trial was carried out with VEE particles expressing the prostate-specific membrane antigen (PSMA) in patients with metastatic castration-resistant prostate cancer [100]. No toxicities were observed after administration of doses of 0.9 × 10^7^ or 0.36 × 10^8^ IU of VEE-PSMA particles. Although no PSMA-specific cellular immune responses or clinical benefit were obtained, generation of neutralizing antibodies indicated that doses used in the study were suboptimal. Liposome encapsulated SFV particles expressing IL-12 have also been subjected to a Phase I clinical study in melanoma and kidney carcinoma patients [101]. Intravenous administration provided five- to 10-fold increase in IL-12 plasma levels, which lasted for 5 days. Due to the encapsulation procedure, the SFV particles were not recognized by the host immune system, which allowed the patients to receive repeated injections. The maximum tolerated dose was determined as 3 × 10^9^ particles per m^2^.

### Update on RNA drugs

Several RNA-based drugs have entered clinical trials [102]. These cover a wide range of delivery methods based on direct injection of siRNA, lipid nanoparticles and encapsulated viral particles. In this context, a Phase I clinical trial with the liposomally encapsulated siRNA targeting protein kinase N3 was evaluated in 34 patients with advanced solid tumors [103]. Ten escalating doses were administered intravenously and the response was monitored by computed tomography/magnetic resonance. The siRNA delivery was well tolerated with only low-grade toxicity. Disease stabilization was observed in 41% of patients with eight individuals showing stable disease with complete or partial metastases regression in some patients. Moreover, Phase I trials are also in progress for cardiovascular and rare liver diseases [104]. A Phase II trial on delivery of RNAi targeting chronic hepatitis B virus (HBV) by lipid nanoparticles is currently in progress for patients with chronic HBV infections [104]. A Phase III trial has been initiated for the treatment of nonarteritic anterior ischemic optic neuropathy with siRNAs targeting caspase 2 mRNA [105]. Efforts have also been dedicated to finding RNA-based precision drugs for lung cancer including long and short noncoding RNAs for diagnostic and therapeutic purposes [106]. The approach to conduct biomarker-driven clinical trials will support improved lung cancer therapy. Moderna has several mRNA based drugs in clinical trials (mainly Phase I) in the area of infectious diseases against influenza, virus, Zika virus and Chikungunya virus [107].

### Update on RNA vaccines

Recent development of RNA-based vaccines has focused on lipid-nanoparticle encapsulation of RNA and applications of self-amplifying RNA virus vectors. For example, nucleoside-modified Zika virus prM and E glycoprotein RNA molecules have been encapsulated in lipid-nanoparticles [108]. A single low-dose intradermal injection elicited potent and durable neutralizing antibodies in mice and nonhuman primates. Protection against challenges with Zika virus was achieved with 30 μg and 50 μg in mice and nonhuman primates, respectively. Self-amplifying RNA vectors such as Kunjin virus [109] and VEE [110] have also provided protection for guinea pigs against challenges with lethal viral doses against Ebola virus. Similarly, vaccination with Kunjin virus particles expressing simian immunodeficiency virus gag-pol made mice resistant to simian immunodeficiency virus challenges [111]. As for RNA drugs, generation of molecularly optimized vaccines by identification of tumor-specific individual mutations in cancer patients has gained momentum [112]. In this context, active personalized cancer vaccines have been subjected to clinical testing. In addition to intranodal administration of synthetic RNA vaccines, second-generation RNA vaccines comprising RNA lipoplex nanoparticle formulations have reached the clinical stage [112]. For instance, the RNA-lipoplex nanoparticles (lipoMERIT) encoding shared tumor antigens for potent melanoma immunotherapy were assessed in a Phase I/II clinical trial [113]. Preliminary data from the on-going study confirmed good safety and tolerability in more than 40 patients. Moreover, a high rate of vaccine-induced immunity was observed and multiple injections of Lipo-MERIT resulted in *de novo* induction of antigen-specific immune responses and potent expansion of pre-existing immunity [113].

## Conclusion & future perspective

RNA-based biopharmaceuticals and vaccines represent a relatively new approach in drug discovery [114]. However, the application range is wide with the potential of both prophylactic and therapeutic interventions for a number of diseases including cancer, diabetes, tuberculosis and cardiovascular and infectious diseases. Although the majority of the 700 DNA- and RNA-based therapeutic drug candidates are in preclinical development and only a limited number in clinical trials, it has been estimated that they will have a market value of $1.2 billion by 2020. Globally, there are some 160 companies and 65 academic teams currently involved in RNA-based therapies [114]. At the time being, at least 12 mRNA vaccines are in development. It also appears that RNA therapeutics have become more promising as potential drugs in comparison to DNA-based drugs. Especially, the development of drugs and vaccines based on mRNA and RNA virus delivery has become attractive options to previously developed olligonucleotide-based therapies.

Today, there are still several issues related to toxicity and drug delivery that needs to be addressed for RNA-based drugs and vaccines, but recent current development related to RNA stability and delivery methods based on encapsulated RNA molecules and self-amplifying RNA viruses will improve the possibility to make this approach a valid alternative for future medicine development

**Executive summary**

- RNA stability has been improved by addition of cap analogs and engineering of polyA tails
- 5′ and 3′ end UTRs and chemical modifications provide improvement on RNA translation and production
- RNA delivery has been optimized by RNA encapsulation and application of self-amplifying RNA viruses
- RNA-based drugs and vaccines have demonstrated therapeutic efficacy in preclinical animal models and in clinical trials

## References

[1] Drews J (2000). Drug discovery: a historical perspective.

[2] Dimitrov DS (2012). Therapeutic proteins.

[3] Chen J, Xie J (2012). Progress on RNAi-based molecular medicines.

[4] Brawerman G (1974). Eukaryotic messenger RNA.

[5] Burgess DJ (2012). RNA stability: remember your driver.

[6] Perche F, Torchilin VP (2013). Recent trends in multifunctional liposomal nanocarriers for enhanced tumor targeting.

[7] Cohn L, Delamarre L (2014). Dendritic cell-targeted vaccines.

[8] Lundstrom K (2016). Self-replicating RNA viral vectors in vaccine development and gene therapy.

[9] Shatkun AJ (1976). Capping of eukaryotic mRNAs.

[10] McNamara MA, Nair SK, Holl EK (2015). RNA-based vaccines in cancer immunotherapy.

[11] Pasquinelli AE, Dahlberg JE, Lund E (1995). Reverse 5′ caps in RNAs made *in vitro* by phage RNA polymerases.

[12] Stepinski J, Waddell C, Stolarski R, Darzynkiewicz E, Rhoads RE (2001). Synthesis and properties of mRNAs containing the novel ‘anti-reverse’ cap analogs 7-methyl(3′-O-methyl)GpppG and 7-methyl(33′-deoxy)GpppG.

[13] Zohra FT, Chowdhury EH, Tada S, Hoshiba T, Akaike T (2007). Effective delivery with enhanced translational activity synergistically accelerates mRNA-based transfection.

[14] Jaschke A, Hofer K, Nubel G, Frindert J (2016). Cap-like structures in bacterial RNA and epitranscriptomic modification.

[15] Cahova H, Winz ML, Hofer K, Nubel G, Jaschke A (2015). NAD captureSeq indicates NAD as a bacterial cap for a subset of regulatory RNAs.

[16] Bird JG, Zhang Y, Tian Y (2016). The mechanism of RNA 5′ capping with NAD+, NADH and dephospho-CoA.

[17] Bernstein P, Peltz SW, Ross J (1989). The poly(A)-poly(A)-binding protein complex is a major determinant of mRNA stability *in vitro*.

[18] Gingras A-C, Raught B, Sonenberg N (1999). eIF4 initiation factors: effectors of mRNA recruitment to ribosomes and regulators of translation.

[19] Holtkamp S, Kreiter S, Selmi A (2006). Modification of antigen-encoding RNA increases stability, translational efficacy, and T cell stimulatory capacity of dendritic cells.

[20] Munroe D, Jacobson A (1990). mRNA Poly(A) tail a 3′ enhancer of translational initiation.

[21] Mockey M, Gonçalves C, Dupuy FP, Lemoine FM, Pichon C, Midoux P (2006). mRNA transfection of dendritic cells: synergistic effect of ARCA mRNA capping with Poly(A) chains in cis and in trans for a high protein expression level.

[22] Tcherepanova IY, Adams MD, Feng X (2008). Ectopic expression of a truncated CD40L protein from synthetic posttranscriptionally capped RNA in dendritic cells induces high levels of IL-12 secretion.

[23] van der Velden AW, Thomas AA (1999). The role of the 5′ untranslated region of an mRNA in translation regulation during development.

[24] Jansen RP (2001). mRNA localization: message on the move.

[25] Bashirullah A, Cooperstock RL, Lipshitz HD (2001). Spatial and temporal control of RNA stability.

[26] Turanov AA, Lobanov AV, Hatfield DL, Gladyshev VN (2013). UGA-position dependent incorporation of selenocysteine into mammalian selenoproteins.

[27] Walczak R, Westhof E, Carbon P, Krol A (1996). A novel RNA structural motif in the selenocysteine insertion element of eukaryotic selenoprotein mRNAs.

[28] Mignone F, Gissi C, Liuni S, Pesole G (2002). Untranslated regions of mRNAs.

[29] Deo RC, Bonanno JB, Sonenberg N, Burley SK (1999). Recognition of polyadenylate RNA by the poly(A)-binding protein.

[30] Zhao Y, Moon E, Carpenito C (2010). Multiple injections of electroporated autologous T cells expressing a chimeric antigen receptor mediate regression of human disseminated tumor.

[31] Kreiter S, Selmi A, Diken M (2010). Intranodal vaccination with naked antigen-encoding RNA elicits potent prophylactic and therapeutic antitumoral immunity.

[32] Kreiter S, Selmi A, Diken M (2008). Increased antigen presentation efficiency by coupling antigens to MHC class I trafficking signals.

[33] Hornung V, Ellegast J, Kim S (2006). 5′-Triphosphate RNA is the ligand for RIG-I.

[34] Kariko K, Buckstein M, Ni H, Weissman D (2005). Suppression of RNA recognition by Toll-like receptors: the impact of nucleoside modification and the evolutionary origin of RNA.

[35] Kariko K, Muramatsu H, Welsh FA (2008). Incorporation of pseudouridine into mRNA yields superior nonimmunogenic vector with increased translational capacity and biological stability.

[36] Kariko K, Muramatsu H, Ludwig J, Weissman D (2011). Generating the optimal mRNA for therapy: HPLC purification eliminates immune activation and improves translation of nucleoside-modified, protein-encoding mRNA.

[37] Wolff JA, Malone RW, Williams P (1990). Direct gene transfer into mouse muscle *in vivo*.

[38] Conry RM, LoBuglio AF, Wright M (1995). Characterization of a messenger RNA polynucleotide vaccine vector.

[39] Steitz J, Britten CM, Wolfel T, Tuting T (2006). Effective induction of anti-melanoma immunity following genetic vaccination with synthetic mRNA coding for the fusion protein EGFP.TRP2.

[40] Fotin-Mleczek M, Duchardt KM, Lorenz C (2011). Messenger RNA-based vaccines with dual activity induce balanced TLR-7 dependent adaptive immune responses and provide antitumor activity.

[41] Houseley J, Tollervey D (2009). The many pathways of RNA degradation.

[42] Probst J, Brechtel S, Scheel B (2006). Characterization of the ribonuclease activity on the skin surface.

[43] Qiu P, Ziegelhoffer P, Sun J, Yang NS (1996). Gene gun delivery of mRNA *in situ* results in efficient transgene expression and genetic immunization.

[44] Dileo J, Miller TE, Chesnoy S, Huang L (2003). Gene transfer to subdermal tissues via a new gene gun design.

[45] Scheel B, Aulwurm S, Probst J (2006). Therapeutic anti-tumor immunity triggered by injections of immunostimulating singlestrandedRNA.

[46] Scheel B, Teufel R, Probst J (2005). Toll-like receptor-dependent activation of several human blood cell types by protamine condensed mRNA.

[47] Sköld AE, van Beek JJ, Sittig SP (2015). Protamine-stabilized RNA as an *ex vivo* stimulant of primary human dendritic cell subsets.

[48] Hoerr I, Obst R, Rammensee HG, Jung G (2000). *In vivo* application of RNA leads to induction of specific cytotoxic T lymphocytes and antibodies.

[49] Weide B, Pascolo S, Scheel B (2009). Direct injection of protamine- protected mRNA: results of a Phase I/II vaccination trial in metastatic melanoma patients.

[50] McKee AS, Marrack P (2017). Old and new adjuvants.

[51] Akasaki Y, Kikuchi T, Irie M (2011). Cotransfection of Poly(I:C) and siRNA of IL-10 into fusions of dendritic and glioma cells enhances antitumor T helper type induction in patients with glioma.

[52] Kreiter S, Diken M, Selmi A, Tureci O, Sahin U (2011). Tumor vaccination using messenger RNA: prospects of a future therapy.

[53] Sahin U, Kariko K, Tureci O (2014). mRNA-based therapeutics – developing a new class of drugs.

[54] Schlake T, Thess A, Fotin-Mleczek M, Kallen K-J (2012). Developing mRNA-vaccine technologies.

[55] Sayour EJ, Sanchez-Perez L, Flores C, Mitchell DA (2015). Bridging infectious disease vaccines with cancer immunotherapy: a role for targeted RNA based immunotherapeutics.

[56] Bettinger T, Read ML (2001). Recent developments in RNA based strategies for cancer gene therapy.

[57] Lu D, Benjamin R, Kim M, Conry RM, Curiel DT (1994). Optimization of methods to achieve mRNA-mediated transfection of tumor cells *in vitro* and *in vivo* employing cationic liposome vectors.

[58] Wasungu L, Hoekstra D (2006). Cationic lipids lipoplexes and intracellular delivery of genes.

[59] Little SR, Lynn DM, Ge Q (2004). Poly-β amino estercontaining microparticles enhance the activity of nonviral genetic vaccines.

[60] Phua KKL, Leong KW, Nair SK (2013). Transfection efficiency and transgene expression kinetics of mRNA delivered in naked and nanoparticle format.

[61] Su X, Fricke J, Kavanagh DG, Irvine DJ (2011). *In vitro* and *in vivo* mRNA delivery using lipid-enveloped pH-responsive polymer nanoparticles.

[62] Phua KKL, Nair SK, Leong KW (2014). Messenger RNA (mRNA) nanoparticle tumour vaccination.

[63] Phua KKL, Staats HF, Leong KW, Nair SK (2014). Intranasal mRNA nanoparticle vaccination induces prophylactic and therapeutic anti-tumor immunity.

[64] Hess PR, Boczkowski D, Nair SK, Snyder D, Gilboa E (2006). Vaccination with mRNAs encoding tumor-associated antigens and granulocyte-macrophage colony-stimulating factor efficiently primes CTL responses, but is insufficient to overcome tolerance to a model tumor/self antigen.

[65] Mockey M, Bourseau E, Chandrashekhar V (2007). mRNA-based cancer vaccine: prevention of B16 melanoma progression and metastasis by systemic injection of MART1 mRNA histidylated lipoplexes.

[66] Perche F, Benvegnu T, Berchel M (2011). Enhancement of dendritic cells transfection *in vivo* and of vaccination against B16F10 melanoma with mannosylated histidylated lipopolyplexes loaded with tumor antigen messenger RNA.

[67] Boczkowski D, Nair SK, Snyder D, Gilboa E (1996). Dendritic cells pulsed with RNA are potent antigen-presenting cells *in vitro* and *in vivo*.

[68] Nair SK, Hull S, Coleman D, Gilboa E, Lyerly HK, Morse MA (1999). Induction of carcinoembryonic antigen (CEA)-specific cytotoxic T-lymphocyte responses *in vitro* using autologous dendritic cells loaded with CEA peptide or CEA RNA in patients with metastatic malignancies expressing CEA.

[69] Ponsaerts P, Van Tendeloo VFI, Berneman ZN (2003). Cancer immunotherapy usingRNA-loaded dendritic cells.

[70] Schuler G, Schuler-Thurner B, Steinman RM (2003). The use of dendritic cells in cancer immunotherapy.

[71] Heiser A, Coleman D, Dannull J (2002). Autologous dendritic cells transfected with prostate-specific antigen RNA stimulate CTL responses against metastatic prostate tumors.

[72] Morse MA, Nair SK, Boczkowski D (2002). The feasibility and safety of immunotherapy with dendritic cells loaded with CEA mRNA following neoadjuvant chemoradiotherapy and resection of pancreatic cancer.

[73] Morse MA, Nair SK, Mosca PJ (2003). Immunotherapy with autologous, human dendritic cells transfected with carcinoembryonic antigen mRNA.

[74] Caruso DA, Orme LM, Neale AM (2004). Results of a phase 1 study utilizing monocyte-derived dendritic cells pulsed with tumor RNA in children and young adults with brain cancer.

[75] Nair SK, Morse M, Boczkowski D (2002). Induction of tumor specific cytotoxic T lymphocytes in cancer patients by autologous tumor RNA-transfected dendritic cells.

[76] Su Z, Dannull J, Heiser A (2003). Immunological and clinical responses in metastatic renal cancer patients vaccinated with tumor RNA-transfected dendritic cells.

[77] Dannull J, Su Z, Rizzieri D (2005). Enhancement of vaccine mediated antitumor immunity in cancer patients after depletion of regulatory T cells.

[78] Bonehill A, Van Nuffel AMT, Corthals J (2009). Single step antigen loading and activation of dendritic cells by mRNA electroporation for the purpose of therapeutic vaccination in melanoma patients.

[79] Kyte JA, Kvalheim G, Aamdal S, Sæbøe-Larssen S, Gaudernack G (2005). Preclinical full-scale evaluation of dendritic cells transfected with autologous tumor-mRNA for melanoma vaccination.

[80] Schuurhuis DH, Verdijk P, Schreibelt G (2009). *In situ* expression of tumor antigens by messenger RNA-electroporated dendritic cells in lymph nodes of melanoma patients.

[81] Strauss JH, Strauss EG (1994). The alphaviruses: gene expression, replication, and evolution.

[82] Pijlman GP, Suhrbier A, Khromykh AA (2006). Kunjin virus replicons: an RNA-based, non-cytopathic viral vector system for protein production, vaccine and gene therapy applications.

[83] Osakada F, Callaway EM (2013). Design and generation of recombinant rabies virus vectors.

[84] An H, Kim GN, Kang CY (2013). Genetically modified VSV(NJ) vector is capable of accommodating a large foreign gene insert and allows high level gene expression.

[85] Radecke F, Spielhofer P, Schneider H (1995). Rescue of measles viruses from cloned DNA.

[86] Pyankov OV, Bodnev SA, Pyankova OG (2015). A Kunjin replicon virus-like vaccine provides protection against Ebola virus infection in nonhuman primates.

[87] Marzi A, Robertson SJ, Haddock E (2015). Ebola vaccine. VSV-EBOV rapidly protects macaques against infection with the 2014/2015 Ebola virus outbreak strain.

[88] Wilson JA, Hart MK (2001). Protection from Ebola virus mediated by cytotoxic T-lymphocytes specific for the viral nucleoprotein.

[89] Huttner A, Dayer JA, Yerly S (2015). The effect of dose on the safety and immunogenicity of the VSV Ebola candidate vaccine: a randomized double-blind, placebo-controlled Phase I/II trial.

[90] Hu HM, Chen HW, Hsiao Y (2016). The successful induction of T-cell and antibody responses by a recombinant measles virus-vectored tetravalent dengue vaccine provides partial protection against dengue-2 infection.

[91] Kamrud KI, Coffield VM, Owens G (2010). *In vitro* and *in vivo* characterization of microRNA-targeted alphavirus replicon and helper RNAs.

[92] Bhomia M, Sharma A, Gayen M (2013). Artificial microRNAs can effectively inhibit replication of Venezuelan equine encephalitis virus.

[93] Hoang-Le D, Smeenk L, Anraku I (2009). A Kunjin replicon vector encoding granulocyte macrophage colony-stimulating factor for intra-tumoral gene therapy.

[94] Garcia-Hernandez ML, Gray A, Hubby B, Klinger OJ, Kast WM (2008). Prostate stem cell antigen vaccination induces a long-term protective immune response against prostate cancer in the absence of autoimmunity.

[95] Martikainen M, Niittykoski M, von und zu Frauenberg M (2015). MicroRNA-attenuated clone of virulent Semliki Forest virus overcomes antiviral type I interferon in resistant mouse CT-2A glioma.

[96] Ying H, Zaks TZ, Wang RF (1999). Cancer therapy using a self-replicating RNA vaccine.

[97] Pepini T, Pulichino AM, Carsillo T (2017). Induction of an IFN-mediated antiviral response by a self-amplifying RNA vaccine: implications for vaccine design.

[98] Démoulins T, Englezou PC, Milona P (2017). Self-replicating RNA vaccine delivery to dendritic cells.

[99] Beissert T, Koste L, Perkovic M (2017). Improvement of *in vivo* expression of genes delivered by self-amplifying RNA using vaccinia virus immune evasion proteins.

[100] Slovin SF, Kehoe M, Durso R (2013). A Phase I dose escalation trial of vaccine replicon particles (VRP) expressing prostate-specific membrane antigen (PSMA) in subjects with prostate cancer.

[101] Lundstrom K, Boulikas T (2002). Breakthrough in cancer therapy: encapsulation of drugs and viruses.

[102] Lundstrom K (2015). RNA-based drugs and vaccines.

[103] Schultheis B, Strumberg D, Santel A (2014). First-in-human phase I study of the liposomal RNA interference therapeutic Atu027 in patients with advanced solid tumors.

[104] Arbutus Biopharma http://www.arbutusbio.com/portfolio/arb-1467-rnai.php.

[105] Quark QPI-1007 http://quarkpharma.com/?page_id=23.

[106] Moderna Therapeutics Pipeline. http://www.modernatx.com/pipeline.

[107] Tian H, Zhou C, Yang J, Li J, Gong Z (2017). Long and short noncoding RNAs in lung cancer precision medicine: Opportunities and challenges.

[108] Pardi N, Hogan MJ, Pelc RS (2017). Zika virus protection by a single low-dose nucleoside-modified mRNA vaccination.

[109] Reynard O, Mokhonov V, Mokhonova E (2011). Kunjin virus replicon-based vaccines expressing Ebola virus glycoprotein GP protect the guinea pig against lethal Ebola virus infection.

[110] Pushko P, Bray M, Ludwig GV (2000). Recombinant RNA replicons derived from attenuated Venezuelan equine encephalitis virus protect guinea pigs and mice from Ebola hemorrhagic fever virus.

[111] Anraku I, Mokhonov VV, Rattanasena P (2008). Kunjin replicon-based simian immunodeficiency virus gag vaccines.

[112] Grabbe S, Haas H, Diken M, Kranz LM, Langguth P, Sahin U (2016). Translating nanoparticulate-personalized cancer vaccines into clinical applications: case study with RNA-lipoplexes for the treatment of melanoma.

[113] Heesen L, Jabulowksy R, Loquai C (7–10 December 2017). A first-in-human Phase I/II clinical trial assessing novel mRNA-lipoplex nanoparticles encoding shared tumor antigens for potent immunotherapy.

[114] Gousseinov E, Kozlov M, Scanlan C (15 September 2015). RNA-based therapeutics and vaccines. http://www.genengnews.com/gen-exclusives/rna-based-therapeutics-and-vaccines/77900520.

